# Facile Synthesis of BiVO_4_@ZIF−8 Composite with Heterojunction Structure for Photocatalytic Wastewater Treatment

**DOI:** 10.3390/ma14237424

**Published:** 2021-12-03

**Authors:** Runjiang Guo, Yurui Xing, Mengqian Liu, Tanglong Bai, Chaodan Pu, Hongti Zhang

**Affiliations:** School of Physical Science and Technology, ShanghaiTech University, Shanghai 201210, China; guorj@shanghaitech.edu.cn (R.G.); xingyr@shanghaitech.edu.cn (Y.X.); liumq@shanghaitech.edu.cn (M.L.); baitl@shanghaitech.edu.cn (T.B.)

**Keywords:** nanorods, metal–organic framework, heterojunction photocatalyst, wastewater treatment

## Abstract

Water pollution has always been a serious problem across the world; therefore, facile pollutant degradation via light irradiation has been an attractive issue in the field of environmental protection. In this study, a type of Zn-based metal–organic framework (ZIF−8)-wrapped BiVO_4_ nanorod (BiVO_4_@ZIF−8) with high efficiency for photocatalytic wastewater treatment was synthesized through a two-step hydrothermal method. The heterojunction structure of BiVO_4_@ZIF−8 was confirmed by morphology characterization. Due to the introduction of mesoporous ZIF−8, the specific surface area reached up to 304.5 m^2^/g, which was hundreds of times larger than that of pure BiVO_4_ nanorods. Furthermore, the band gap of BiVO_4_@ZIF−8 was narrowed down to 2.35 eV, which enabled its more efficient utilization of visible light. After irradiation under visible light for about 40 min, about 80% of rhodamine B (RhB) was degraded, which was much faster than using pure BiVO_4_ or other BiVO_4_-based photocatalysts. The synergistic photocatalysis mechanism of BiVO_4_@ZIF−8 is also discussed. This study might offer new pathways for effective degradation of wastewater through facile design of novel photocatalysts.

## 1. Introduction

Recently, water purifying technology has received widespread attention as an emerging field. In particular, photocatalytic degradation is recognized as the most promising way to purify wastewater, due to its low cost and environmentally friendly properties [1,2,3,4]. Photocatalysts can degrade noxious organic pollutants under the irradiation of sunlight without producing any toxic remains [5,6]. The key to realizing this advantage relies on precise design of the photocatalysts.

Metal oxide semiconductors, such as TiO_2_ (P25), ZnO, Bi_2_O_3_, BiFeO_3_, ZnSnO_3_ and BiVO_4_, have all been proved to be efficient photocatalysts [7,8,9,10,11,12,13,14]. Among the many currently studied photocatalysts, BiVO_4_, as a cost-effective, eco-friendly and chemically stable material has garnered considerable interest recently. As indicated in previous studies, monoclinic scheelite, tetragonal zircon and tetragonal scheelite are the most common forms of BiVO_4_ existing in nature [15,16]. Compared with tetragonal BiVO_4_, which mainly responds to UV light, BiVO_4_ with a monoclinic scheelite structure has better photon harvesting and more sensitive light response properties due to its relatively narrower band gap (2.4 eV); therefore, m-BiVO_4_ can generate electron–hole pairs under the irradiation of visible light [17,18]. Recently, many efforts have been made to enhance the photocatalytic properties of BiVO_4_. Guo et al. synthesized V^4+^ self-doped BiVO_4_ nanorods with [010] oriented for water purification. In these nanorods, the oxygen vacancies and V^4+^ ions could act as charge carrier traps and adsorption sites, thus inhibiting the recombination of photogenerated electron–hole pairs, and resulting in an excellent photocatalyst [19].

Photocatalysts’ size and shape can also influence their degradation efficiency. Currently, mainstream photocatalysts are often in a solid bulk shape. However, bulk-shaped photocatalysts always suffer from an increased electron–hole recombination rate, poor electron transportation mobility and worse surface absorbability [20,21]. Therefore, investigating photocatalysts with mesoporous structures has attracted a significant amount of attention. Compared with solid photocatalysts, photocatalysts with mesoporous structures have a relatively larger specific surface area. With the enlarged specific surface area, there will be more contact between organic dyes and the catalyst, thus improving the absorption and photodegradation process [22,23]. Metal–organic frameworks (MOFs), as a type of superior mesoporous material, could be prominent host candidates for water splitting due to their high surface area, mesoporous structure, tunable shape and chemical stability [24]. There are a large number of organic ligands around central metal ions, and the chemical bonds between the organic ligands and metal ions are also flexible. Therefore, MOFs can absorb photons and transfer electrons to the metal ions easily. In addition, the synthesis processes for MOFs are convenient. MOFs are usually synthesized by mixing the aromatic multicarboxylatic ligands and metal ions in organic solutions, which is much more convenient than other synthesis methods. Thus, MOFs can be prominent candidates for photodegradation [25,26,27].

Photocatalysts with a single component readily suffer from rapid electron–hole recombination. Therefore, incorporating metal oxide semiconductors and MOFs together might form new types of photocatalysts with improved photocatalytic efficiency by combining their advantages. In addition, to take advantage of the extremely large surface area of MOFs, which can provide more active sites for dye degradation, a semiconductor–MOF heterojunction can be formed at the interface; this can generate an in-built electric field and inhibit rapid electron–hole recombination, thus enhancing their life time and improving photocatalytic efficiency [28,29]. Moreover, the heterojunction can change the distribution of photogenerated electron–hole pairs in order to adjust the band gap width and promote photocatalytic performance [30].

In this research, ZIF−8-wrapped BiVO_4_ nanorods with a narrower band gap and larger specific surface area were fabricated through hydrothermal and self-sedimentation processes. The photocatalytic performance of BiVO_4_@ZIF−8 was evaluated by the degradation of RhB solutions. With the newly designed composites, about 70% of the organic dye could be degraded under irradiation with visible light for nearly 40 min. The mechanism of this synergistic photocatalysis was also investigated.

## 2. Materials and Methods

### 2.1. Material Synthesis

#### 2.1.1. Material Preparation

Bismuth nitrate pentahydrate (Bi(NO_3_)_3_·5H_2_O), zinc nitrate hexahydrate (Zn(NO_3_)_2_·6H_2_O), sodium oleate (C_17_H_33_CO_2_Na ≥ 98%), ammonium vanadate (NH_4_VO_3_ ≥ 98%), ammonium hydroxide and nitric acid were all bought from Sinopharm Chemical Reagent Co., Ltd. 2-Methylimidazole (C_4_H_6_N_2_ ≥ 98%) was purchased from Macklin Inc.

#### 2.1.2. Synthesis of BiVO_4_ Nanorods

BiVO_4_@ZIF−8 was synthesized via a two-step method. Typically, the BiVO_4_ precursor was synthesized via hydrothermal reaction first, and then the final BiVO_4_@ZIF−8 product was obtained via a self-sedimentation method. In detail, about 0.4 mmol (0.388 g) of bismuth nitrate pentahydrate was dispersed into 20 mL of deionized water under constant stirring. In order to obtain a homogeneous transparent solution, several drops of HNO_3_ were added to the mixture. We defined this solution as A. Likewise, 0.4 mmol (0.096 g) NH_4_VO_3_ and 1 mmol (0.36 g) sodium oleate were dissolved in deionized water, giving solutions which were defined as B and C, respectively. After 30 min magnetic stirring, we mixed solutions A, B and C together to form a yellow homogeneous suspension. Several drops of ammonium hydroxide were then dropped into the suspension to keep it weakly alkaline (pH ≈ 9). Afterwards, the suspension was poured into a PTFE-made hydrothermal reactor (Yu hua Tech Co., Ltd. Shanghai, China), to be kept at 180 °C for 24 h. After the reaction, the brown powder was collected by centrifugation and dried at 80 °C. To enhance its crystallinity, the BiVO_4_ powder was further annealed in a furnace at 450 °C for 2 h.

The second step was to obtain ZIF−8-wrapped BiVO_4,_ named BiVO_4_@ZIF−8, via a self-sedimentation method. Typically, we dissolved 0.5 g Zn(NO_3_)_2_·6H_2_O and 0.8 g 2-methylimidazole separately into absolute methanol to form homogeneous solutions. Then, 1 g as-prepared BiVO_4_ was dispersed into Zn(NO_3_)_2_ solution under vigorous stirring for half an hour. Afterwards, we mixed them together to form a yellow homogeneous suspension and let it stand for half a day. After the reaction, the light yellow powder was collected by centrifugation and dried at 80 °C. The final product was a light yellow BiVO_4_@ZIF−8 composite.

### 2.2. Material Characterization

A JSM-IT500HR/LA instrument (JEOL, Tokyo, Japan) was used for scanning electron microscopy (SEM); a JEM-2100Plus (JEOL, Tokyo, Japan) was used as a transmission electron microscope (TEM); and X-ray diffraction (XRD, D2 Advance, BRUKER, Karlsruhe, Germany) and X-ray photoelectron spectroscopy (XPS, ESCALAB 250Xi, Thermo Fisher Scientific, London, UK) were used to investigate the phase and element information of the final products. Belsorp-MAXII (MicrotracBEL, Osaka, Japan) was used to measure the BET surface area. In this study, the photoluminescence spectra were acquired using a photoluminescence spectrometer (Fluorolog-3 Horiba Scientific, NJ, USA), with 360 nm excitation light. Ultraviolet–visible (UV–Vis) spectra were recorded by a Cary 5000 spectrophotometer (Agilent Technologies, Penang, Malaysia).

### 2.3. Photocatalytic Activity Evaluation

Rhodamine B (RhB) was used to assess the photocatalytic activity of the as-prepared catalyst. Typically, we dispersed 100 mg catalyst (pure BiVO_4_ or BiVO_4_@ZIF in this work) into RhB solution with a concentration of 0.5 mg/50 mL. Before the degradation reaction, the suspension was kept in a dark environment for 30 min to reach a photoequilibrium state, using ultrasonic vibration. In the catalysis process, we collected the suspension every 10 min via centrifugation. As the characteristic absorption peak for RhB is at 550 nm, the intensity of the absorption peak for RhB was measured by UV–Vis absorption spectra. Equation (1) below was applied to determine the RhB degradation efficiency (the ratio of the remaining RhB concentration and the initial RhB concentration) in the degradation process:

(1) $$\eta_{\mathrm{eff}}=(1-\frac{A_{t}}{A_{0}})\times 100\%$$

where *η_eff_* is the degradation efficiency of RhB, and *A_0_* and *A_t_* are the intensities of absorption peaks before and after degradation for a certain time interval, respectively.

## 3. Results and Discussion

**Figure 1:**
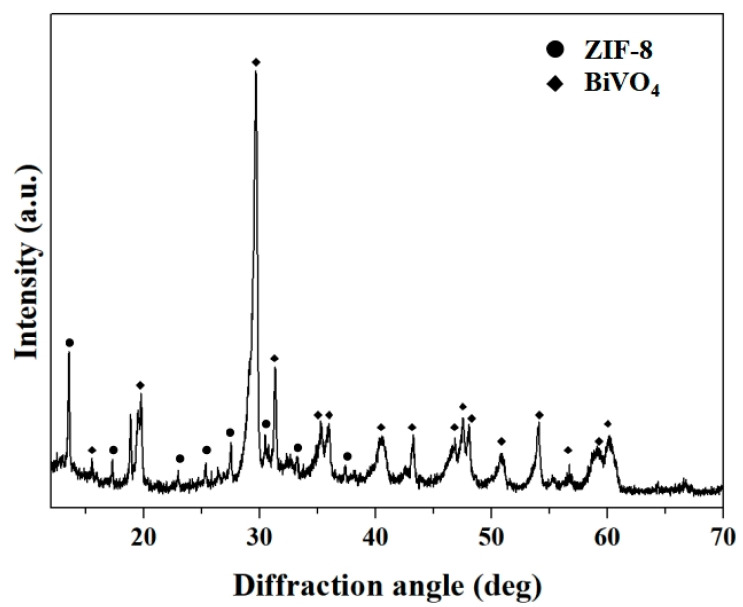
The XRD pattern of BiVO_4_@ZIF−8. The diffraction peaks are assigned to BiVO_4_ and ZIF−8 as indicated.

The phase and crystal structure information of the as-synthesized BiVO_4_ nanorods and BiVO_4_@ZIF-8 composites was examined by XRD analysis, as shown in Figure S1 (which can be found in the Supplementary Materials) and Figure 1, respectively. In Figure S1, all of the diffraction peaks were assigned to the standard monoclinic-type BiVO_4_ (JCPDS card no. 14-0688; a = 5.195 Å, b = 11.704 Å, c = 5.092 Å; space group: I2/a) [31]. There was a minor diffraction peak at 15.11° in the XRD pattern of BiVO_4_, which could distinguish m-BiVO_4_ from tetragonal scheelite BiVO_4_ (t-BiVO_4_) [32]. It can also be seen in Figure S1 that the intensities of the diffraction peaks of m-BiVO_4_ were very strong, suggesting that the defect intensity of BiVO_4_ nanorods was notably reduced via calcination. The lower number of defects and high crystallinity of BiVO_4_ are conducive to its enhanced catalytic performance, since defects in crystal lattices can play the role of combining centers for photogenerated electrons and holes [33,34,35]. In addition, there were two diffraction peaks of BiVO_4_ assigned to (200) and (002) at about 35°, which are characteristic of monoclinic scheelite-type BiVO_4_. The curve in Figure 1 shows the XRD pattern of BiVO_4_@ZIF−8. The majority of diffraction peaks of ZIF−8 were clearly seen between 10° and 30°, indicating the co-existence of BiVO_4_ and ZIF−8. We conclude that the BiVO_4_@ZIF−8 composites were formed simply by the attachment of ZIF−8 nanoparticles to the surface of BiVO_4_ nanorods, and that no compound was formed.

**Figure 2:**
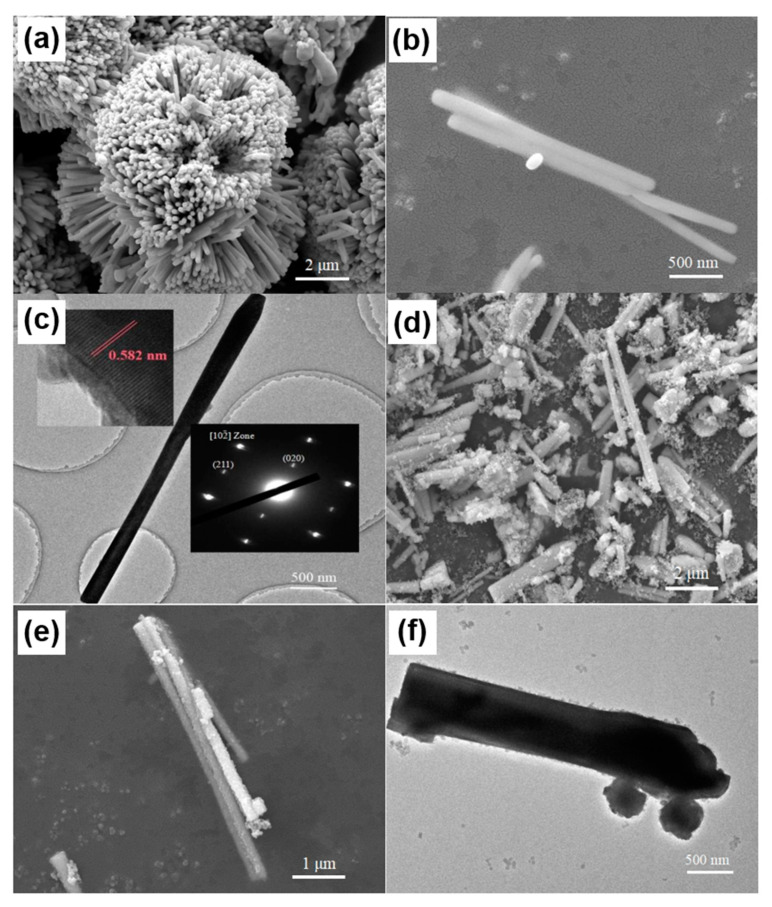
Morphology and structure characterization of BiVO_4_ and BiVO_4_@ZIF−8. Typical SEM images of as-synthesized BiVO_4_ precursor at (**a**) low and (**b**) high magnification; typical (**c**) TEM image with HR-TEM and SAED images inserted of as-synthesized BiVO_4_ precursor with the length of ~3000 nm and width of ~150 nm; typical SEM images of as-synthesized BiVO_4_@ZIF−8 at (**d**) low and (**e**) high magnification; (**f**) typical TEM image of as-synthesized BiVO_4_@ZIF−8 with the ZIF−8 nanoparticles tightly attached to the surface of the BiVO_4_ nanorod.

Morphologies of as-synthesized BiVO_4_ nanorods and BiVO_4_@ZIF−8 composites were obtained by SEM and TEM and are shown in Figure 2a–f. As indicated in Figure 2a–c, the diameter and length of BiVO_4_ nanorods were about 150 nm and 3000 nm, respectively. In addition, the BiVO_4_ nanorods were aggregated together to form a flower-like shape. Figure 2c (insert) shows the HR-TEM image of an individual BiVO_4_ nanorod and its corresponding SAED pattern. As shown in Figure 2c (insert), the diffraction fringes of BiVO_4_ nanorods with the spacing of 0.582 nm were well assigned to their (020) lattice planes (b = 11.704Å, two times the fringe spacing), indicating that the BiVO_4_ nanorods were probably growing along the [010] direction. Figure 2c (insert) also shows the diffraction spots along the [102] zone axis in the SAED pattern. There were two spots around the (000) spots, indexed as the (020) and (211) lattice planes. In addition, there was only one set of diffraction spots in our SAED pattern, indicating that the as-synthesized BiVO_4_ was a pure phase without any impurities. Figure 2d–f show SEM and TEM images of BiVO_4_@ ZIF−8. The surface of BiVO_4_@ZIF−8 nanorods was coarser than that of pure BiVO_4_. In addition, the BiVO_4_@ZIF−8 nanorods were well dispersed, owing to the existence of the MOF. We could clearly see that ZIF−8 nanoparticles with a size of ~30 nm were tightly attached to the BiVO_4_ nanorod’s surface and that a heterojunction between the BiVO_4_ and ZIF−8 was formed, which could enhance the photocatalysis performance of BiVO_4_@ ZIF−8.

**Figure 3:**
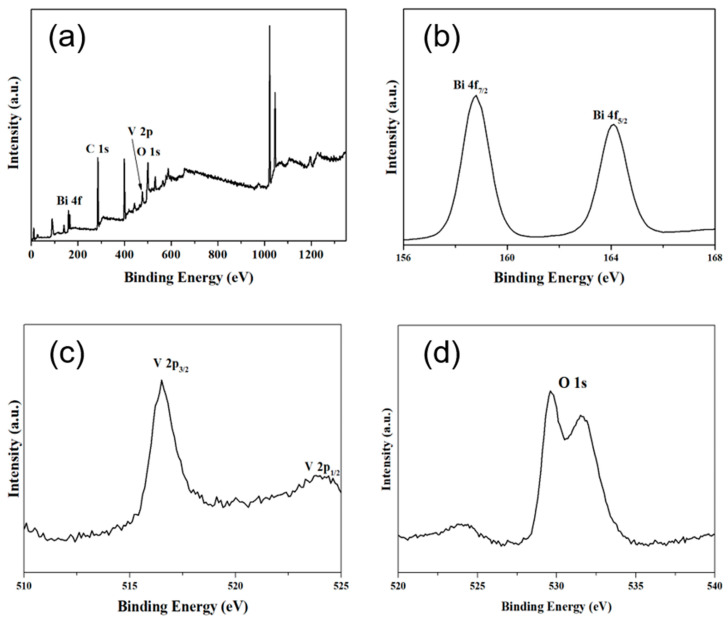
(**a**) XPS spectra of BiVO_4_ showing the presence of Bi, C, O and V. High-resolution XPS spectra showing: (**b**) Bi 4f at 158.5 and 164.4 eV, (**c**) V 2p 516.5 eV and 524.3 eV and (**d**) O 1s at 528.9 and 532.5 eV.

XPS (seen in Figure 3a) showed that only Bi, V, C and O existed in as-prepared BiVO_4_. Figure 3b,c show the characteristic peaks for Bi^3+^ and V^5+^ in BiVO_4_. The binding energies of Bi 4f_7/2_ and Bi 4f_5/2_ were 158.5 and 164.4 eV, respectively, and the binding energies of V 2p_3/2_ and V 2p_1/2_ were 516.5 eV and 524.3 eV, respectively, consistent with previous reports [36,37]. The appearance of the C 1s signal at 284.8 eV might be an instrument error during the XPS test. It is noticeable that, in Figure 3d, the O 1s peaks were split into two peaks, which correspond to the O element in the BiVO_4_ crystal lattice (528.9 eV) and O_2_ absorbed via BiVO_4_ (532.5 eV).

**Figure 4:**
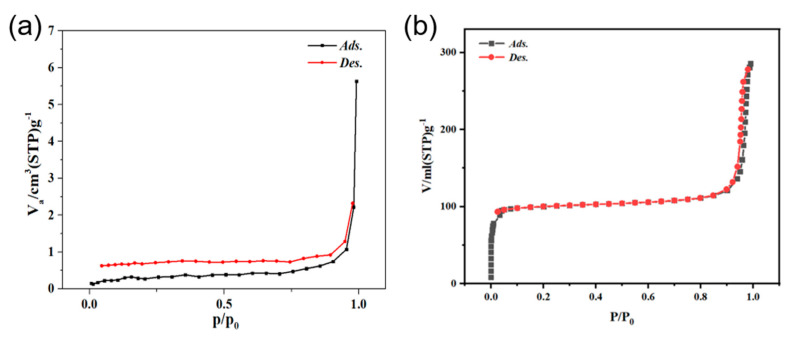
(**a**) The N_2_ adsorption−desorption isotherm of BiVO_4_ nanorods; (**b**) the N_2_ adsorption−desorption isotherm of BiVO_4_@ZIF−8.

Specific surface areas of BiVO_4_ and BiVO_4_@ ZIF−8 were obtained using nitrogen adsorption–desorption tests. Owing to the addition of mesoporous ZIF−8, the as-prepared BiVO_4_@ZIF−8 composite showed a type IV hysteresis loop, as seen in Figure 4b [20]. Moreover, as calculated via the instrument, the surface area of BiVO4@ZIF−8 was as high as 304.5 m^2^/g, which was much larger than that of pure BiVO_4_ (2.53 m^2^/g). Owing to its larger surface area, BiVO_4_@ZIF−8 had more active sites to absorb and degrade organic dyes than pure BiVO_4_; thus, the catalytic performance could be significantly enhanced.

**Figure 5:**
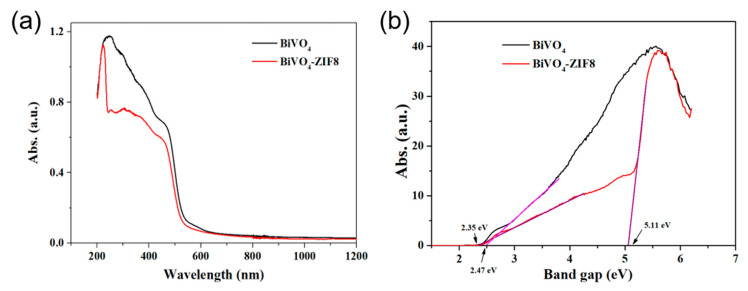
(**a**) The UV–Vis absorption curves of pure BiVO_4_ (black) and BiVO_4_@ZIF−8 (red); (**b**) the corresponding Kubelka–Munk (K–M) transformed curves of pure BiVO_4_ (black) and BiVO_4_@ZIF−8 (red). The band gaps for BiVO_4_@ZIF−8 and pure BiVO_4_ were calculated as 2.35 eV and 2.47 eV, respectively.

The property of light absorption was another crucial factor that affected the photocatalytic properties of BiVO_4_ and BiVO_4_@ZIF−8. Therefore, ultraviolet–visible absorption spectra were applied to characterize the light absorption abilities of BiVO_4_ and BiVO_4_@ZIF−8. From the spectra in Figure 5, we can see that the absorption edges of pure BiVO_4_ and BiVO_4_@ZIF−8 composites were all around 500 nm. Tauc’s equation, as shown in Equation (2), was applied to determine the band gap value of semiconductors with a direct band gap:

(2) $$\alpha hv=A{\left(hv-E_{g}\right)}^{\frac{1}{2}}$$

where *h, v, A, E_g_* and *α* represent Plank’s constant, light frequency, absorbance, band gap value and absorption coefficient, respectively. In Equation (2), the parameters *A* and *α* are constant for a specific sample.

Figure 5b shows the K–M transformed reflectance spectra corresponding to Figure 5a. Extrapolating the quasi-straight line to intersect with the X-axis can provide the band gap values of the measured samples. As calculated in Figure 5b, BiVO_4_ without modification showed a band gap value of 2.47 eV. However, as shown in Figure 5b, after modification with ZIF−8, the band gap value for BiVO_4_@ZIF−8 was divided into two values: 2.35 eV and 5.11 eV. The latter was assigned to the value for ZIF−8. In addition, the band gap value for BiVO_4_ after modification with ZIF−8 (2.35 eV) was slightly narrower than that of pure BiVO_4_ in our research and other papers [31,32,38,39]. Smaller band gaps are typically more favorable for efficient utilization of solar energy to produce photogenerated electron–hole pairs, which could directly enhance the photocatalytic properties.

**Figure 6:**
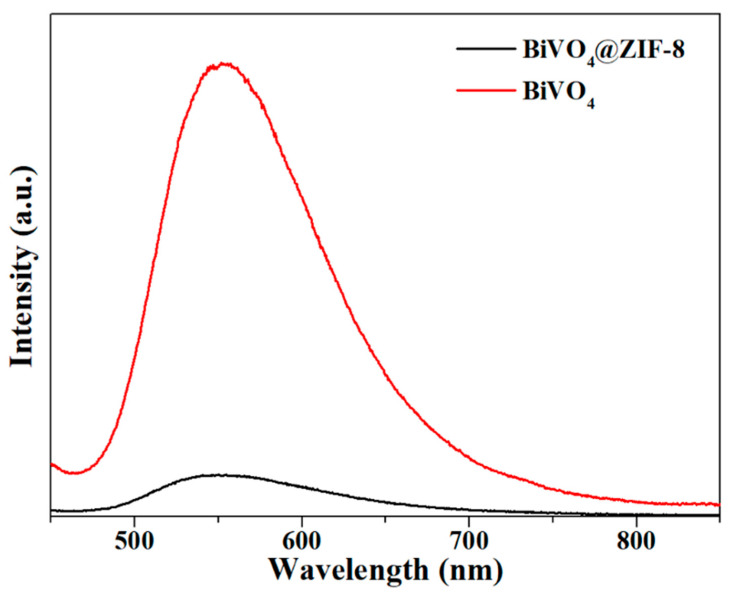
The PL spectrum of BiVO_4_ and BiVO_4_@ZIF−8; the PL intensity of BiVO_4_@ZIF−8 is much weaker than that for pure BiVO_4_.

When the photogenerated electrons and holes are recombining, strong photoluminescence signals will occur and can be detected via PL spectra, as shown in Figure 6. The PL spectra of BiVO_4_ and BiVO_4_@ZIF−8 were both excited at 365 nm, which corresponded to a photon energy of 3.39 eV, and was larger than the band gap of both BiVO_4_ (2.47 eV) and BiVO_4_@ZIF−8 (2.35 eV). This energy was strong enough for both BiVO_4_ and BiVO_4_@ZIF−8 to excite valence electrons to the conduction band. As shown in Figure 6, the specific peak intensities of BiVO_4_@ZIF−8 at 540 nm were much weaker than those of pure BiVO_4_. The weaker PL intensity indicated that the recombination of photogenerated electrons and holes was largely suppressed in the BiVO_4_@ZIF−8 composite, which was conducive to enhancing photocatalytic performance [40].

**Figure 7:**
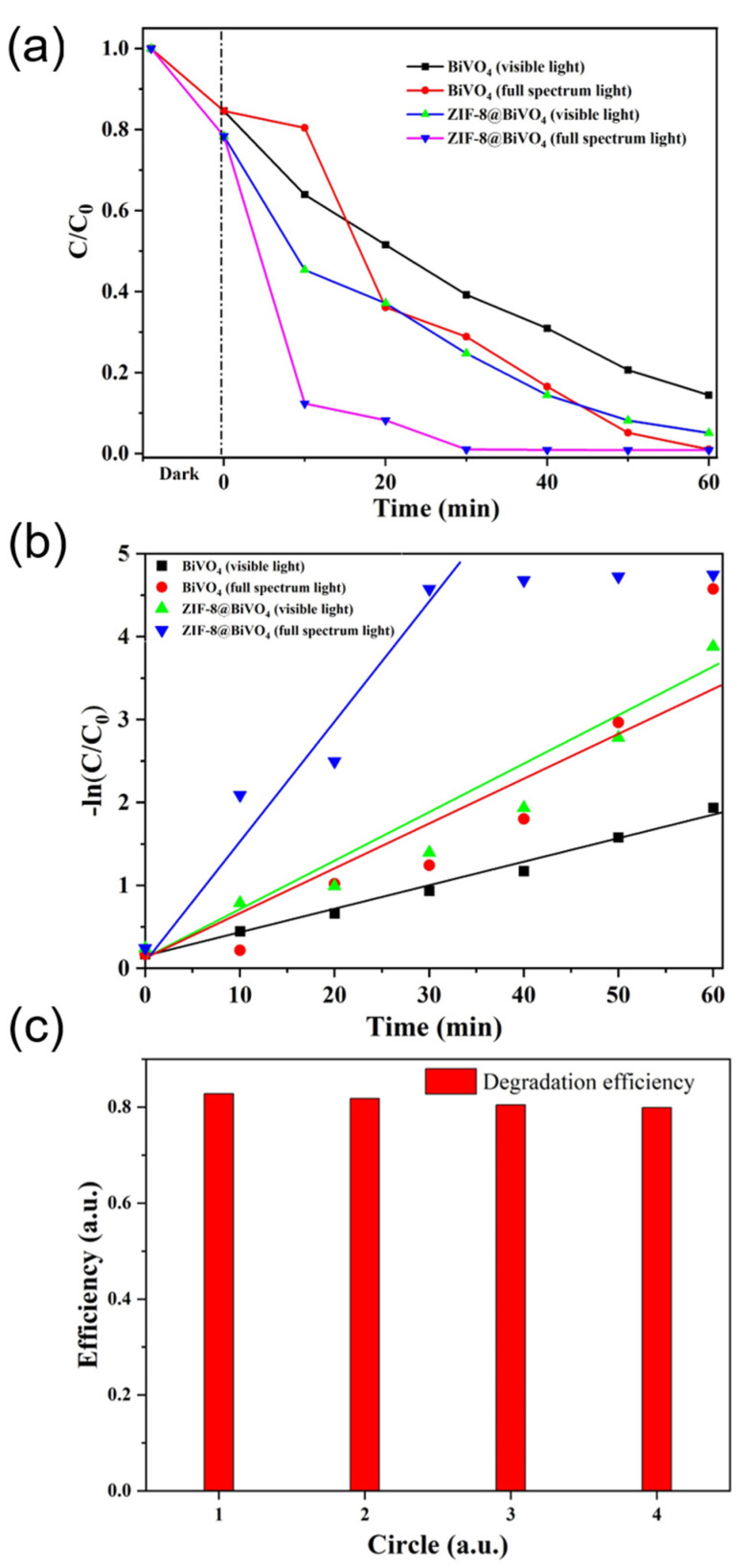
(**a**) The photodegradation efficiency of BiVO_4_ and BiVO_4_@ZIF−8 under full-spectrum and visible light. In the presence of BiVO_4_-ZIF 8, more than 90% of RhB is degraded after 60 min of visible light irradiation, while only 80% of RhB is degraded in the presence of pure BiVO_4_. (**b**) The value of ln (C_0_/C) vs. time for RhB degradation; BiVO_4_@ZIF−8 shows a higher kinetic constant with the value of 0.133 min^−1^. (**c**) The reproducibility test of photocatalysis.

The photocatalytic performance of pure BiVO_4_ nanorods and BiVO_4_@ZIF−8 composites was evaluated by RhB photodegradation tests, as shown in Figure 7. Figure 7a shows the RhB degradation efficiencies of pure BiVO_4_ nanorods and BiVO_4_@ZIF−8 composites under UV light and visible light at different degradation times. As shown in Equation (1), the degradation efficiency of RhB was determined via UV–Vis absorption spectra, as presented in Figure S2. It is noticeable that about 15% to 23% of RhB was missing before the light irradiation of pure BiVO_4_ and BiVO_4_@ZIF−8, respectively, mainly due to the adsorption characteristics of BiVO_4_ and BiVO_4_@ZIF−8. Owing to the larger surface area, more RhB will be absorbed by BiVO_4_@ZIF−8 than by pure BiVO_4_. When RhB is dissolved in water, it is positively charged. Therefore, RhB cations will be attracted by the O^2−^ or OH^−^ anions at the photocatalyst’s surface. As shown in Figure 7a, after being irradiated under visible light for 40 min, about 80% of RhB was degraded by BiVO_4_@ZIF−8, while 32% of RhB still remained with the pure BiVO_4_ nanorods. It can also be seen in Figure 7a that, under full-spectrum irradiation, more than 90% of RhB was degraded by BiVO_4_@ZIF−8 in 20 min and entirely decomposed after 60 min. In comparison, after being irradiated under UV light for 20 min, less than 70% of RhB was degraded by the pure BiVO_4_ nanorods, and still 20% of RhB remained after irradiation for 40 min. The photocatalytic property of pure ZIF−8 is also shown in Figure S2e,f, which show that over 60% of RhB was absorbed by the MOF, owing to its large surface area and mesoporous structure. However, only about 80% of RhB was degraded during the following photodegradation.

At a relatively low concentration (10 mg/L) of RhB solution, the kinetics of the degradation process of RhB could be defined as the pseudo-first-order reaction mode, which means that the values of the logarithm of the concentration ratio (ln(C_0_/C)) and irradiation time have a linear relationship [41]. Therefore, the kinetic constant of k for RhB degradation was defined as the slope of the quasi-line of the logarithm of the concentration ratio and irradiation time, as illustrated in Figure 7b. After calculation, the value of k for BiVO_4_ was 0.05 min^−1^. The k value for BiVO_4_@ZIF−8 reached 0.133 min^−1^, which indicates that BiVO_4_@ZIF−8 showed better photocatalytic activity under solar irradiation than pure BiVO_4_. Being as important as the catalytic efficiency, excellent reproducibility is also a crucial factor for photocatalysts. In Figure 7c, the reproducibility of BiVO_4_@ZIF−8 was examined by recycling BiVO_4_@ZIF−8 for four cycles. The results show that the degradation efficiency of BiVO_4_@ZIF−8 was stable with no drastic deactivation after recycling, indicating its feasibility for practical applications such as water purification.

**Figure 8:**
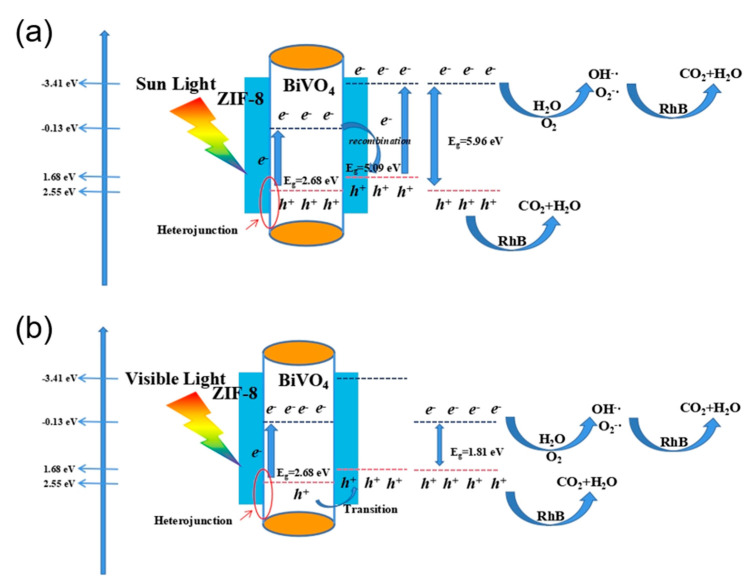
The working mechanism of RhB photodegradation under (**a**) sunlight and (**b**) visible light.

Figure 8 shows the photocatalysis mechanism in the presence of pure BiVO_4_ and BiVO_4_@ZIF−8. As a type of semiconductor, the valence band (VB) is full of electrons, while the conduction band (CB) lacks electrons. When a semiconductor is irradiated under light with energy no less than the E_g_ of that semiconductor, the light can excite the electrons at the VB to the CB. Therefore, holes will be left in the VB, and thus photogenerated electron–hole pairs will be formed. The photogenerated electron–hole pairs can oxidize H_2_O molecules, OH^−^ ions and dissolved O_2_ in H_2_O to form ·OH or O_2_^−^**·** radicals, which have strong oxidation properties. Moreover, the organic dye RhB, with its characteristic absorption wavelength of about 550 nm, could be photosensitized under light as well. Therefore, the sensitized RhB dye could be oxidized into water and CO_2_ by **·**OH or O_2_^−^· radicals, which is one of the possible degradation mechanisms of an organic dye. We may summarize the set of RhB degradation processes as shown in Equations (3)–(6) below [42]:

(3) $$OH^{-}+h^{+}\to \cdot OH$$

(4) $$H_{2}O+h^{+}\to \cdot OH+H^{+}$$

(5) $$e^{-}+O_{2}\to O_{2}^{-}\cdot$$

(6) $$OH/e^{-}/O_{2}^{-}+\mathrm{RhB}\to CO_{2}+H_{2}O$$

In Equation (6), there are two pathways involved in RhB degradation: the stepwise de-ethylation process, and cleavage of the conjugated hydrocarbon structure [43,44], which can be directly seen in the UV–Vis absorption curve in Figure S2. The cleavage of the conjugated hydrocarbon structure leads to a decreased absorption intensity of the RhB solution, and the de-ethylation process leads to the red shift in the absorption peak of RhB to about 500 nm.

The mechanism of the enhanced photocatalysis performance of BiVO_4_@ZIF−8 could be further explained by its band gap structure. The positions of the CB and VB for BiVO_4_ and ZIF−8 are clearly shown in Figure 8. For the BiVO_4_@ZIF−8 heterojunction structure, the VB and CB positions of BiVO_4_ are all much more positive than those of ZIF−8 (the CB and VB positions are −0.13 eV and 2.55 eV for BiVO_4,_ respectively, and −3.41 eV and 1.68 eV for ZIF−8, respectively). Hence, we may discuss the enhancing mechanism under both UV light and visible light. When irradiated under UV light, the photogenerated electron–hole pairs can be generated from both BiVO_4_ and ZIF−8 at the CB and VB, respectively. Owing to the closer position of the CB of BiVO_4_ and the VB of ZIF−8, the electron at the conduction band of BiVO_4_ and the hole at the valence band of ZIF−8 tend to recombine with each other more easily. Therefore, the e^-^ and h^+^ of BiVO_4_@ZIF−8 only remain at the CB of ZIF−8 and the VB of BiVO_4_, respectively, which indicates that the band gap width of BiVO_4_@ZIF−8 is extended to 5.96 eV. The O_2_^−^· at the CB of ZIF−8 has higher energy than that of BiVO_4_; therefore, BiVO_4_@ZIF−8 is more beneficial for RhB degradation. When irradiated under visible light, due to the larger band gap of ZIF−8, the photogenerated electron–hole pairs can only be excited at the BiVO_4_ surface. As the VB of ZIF−8 is more negative than that of BiVO_4_, the covalent Bi-O-Zn at the heterojunction could play a role that transfers the holes at the VB of BiVO_4_ to the VB of ZIF−8 and hinders the recombination of the photogenerated electron–hole pairs [45]. In addition, the processes of degradation often occur at the surfaces of photocatalysts; in the BiVO_4_@ZIF−8 composite, ZIF−8, with its mesoporous structure and large surface area, has more active sites for h^+^ to form free radicals to photodegrade RhB. Thus, BiVO_4_@ZIF−8, with its larger surface area and heterojunction structures, exhibited better photocatalysis performance.

As a class of multiple functional materials, BiVO_4_ and its composites have been widely applied in the field of water treatment via photodegradation. Therefore, we investigated the degradation efficiency via photocatalysis of different photocatalysts based on BiVO_4_ composite materials, as listed in Table S1. In addition, we investigated the performance of different photocatalysts based on ZIF−8 composite materials, as illustrated in Table S2. As shown in Table S2, the BiVO_4_@ZIF−8 heterojunction had a better catalysis performance than other BiVO_4_- or ZIF−8-based composite materials.

## 4. Conclusions

In conclusion, BiVO_4_@ZIF−8, with its larger surface area and heterojunction structure, was fabricated using a hydrothermal technique first, and then a self-sedimentation technique. Owing to its mesoporous surface and heterojunction structure, the as-synthesized BiVO_4_@ZIF−8 exhibited a higher degradation efficiency than that of pure BiVO_4_. As shown in our research, about 90% of RhB was degraded under 60 min of visible light irradiation. The high degradation efficiency and reproducibility demonstrate that BiVO_4_@ZIF−8, as designed in our work, could be a prominent candidate for wastewater treatment.

## Data Availability

The data supporting reported results of the current study are available from the corresponding authors on reasonable request.

## Supplementary Materials

The following are available online at https://www.mdpi.com/article/10.3390/ma14237424/s1, Figure S1: The XRD pattern (a) and the standard PDF card (b) of pure BiVO_4_. Figure S2: The light absorption spectra of RhB solution after photo-degraded by BiVO_4_ (a) and BiVO_4_@ZIF-8 (b) in visible light; BiVO_4_ (c), BiVO_4_@ZIF-8 (d) and pure ZIF-8 (e) in UV light; (f) The photo degradation efficiency of pure ZIF-8 under UV light. Table S1: The performances of photocatalysts based on different BiVO_4_ composite materials. Table S2: The performances of photocatalysts based on different MOF composite materials.
