# Supplementary material for: Facile Synthesis of BiVO4@ZIF−8 Composite with Heterojunction Structure for Photocatalytic Wastewater Treatment
Source: Materials (Basel). 2021 Dec 3;14(23):7424. doi: 10.3390/ma14237424 (PMC8658979; doi:10.3390/ma14237424)
Supplement: Supplementary file 1 [file materials-14-07424-s001.zip › materials-1457768-supplementary.pdf]

Supplementary Materials

Supplementary Materials sections were cited in the main text as Figures S1 and S2, Tables S1 and S2.

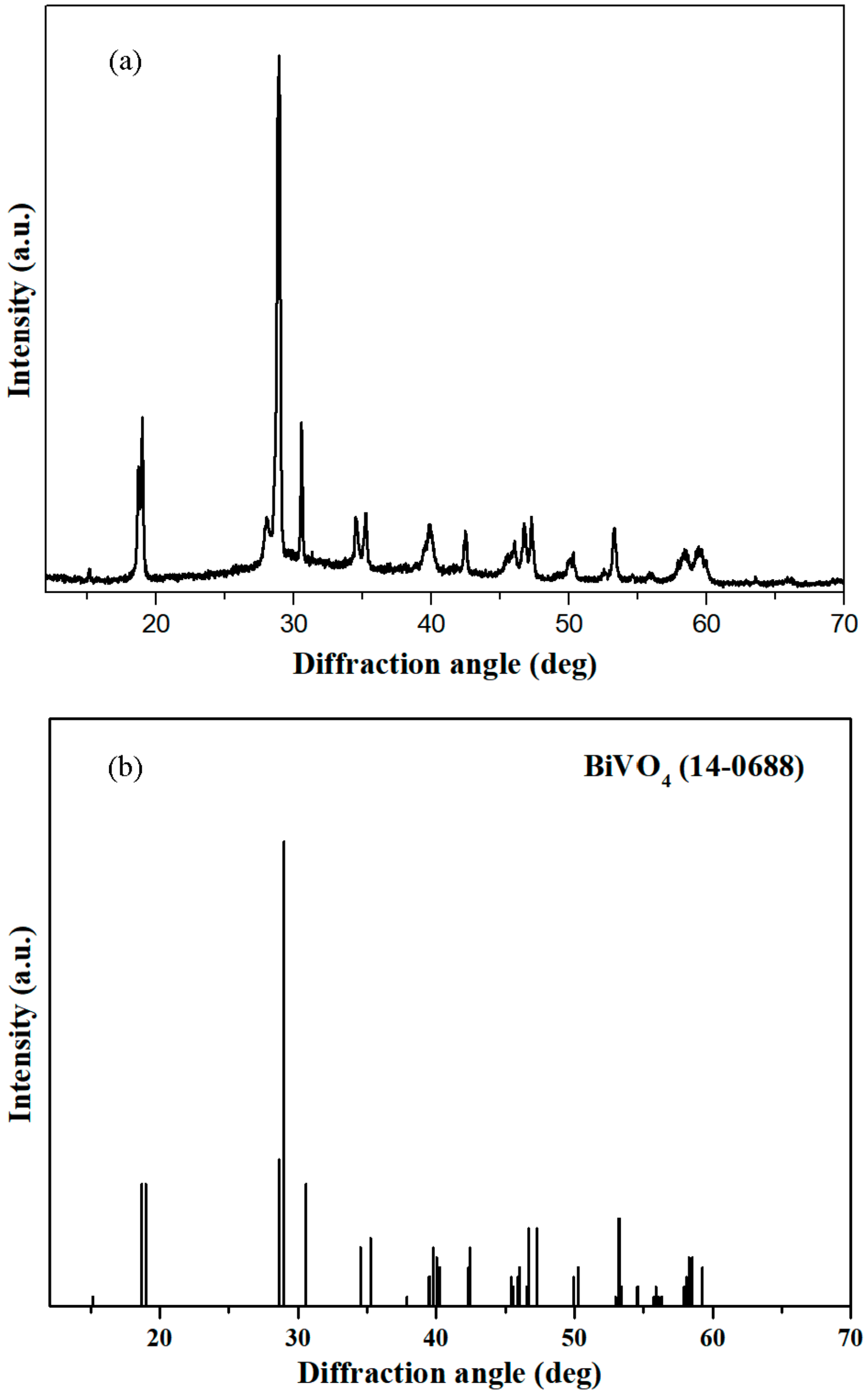

Figure S1. The XRD pattern (**a**) and the standard PDF card (**b**) of pure $BiVO_4$.

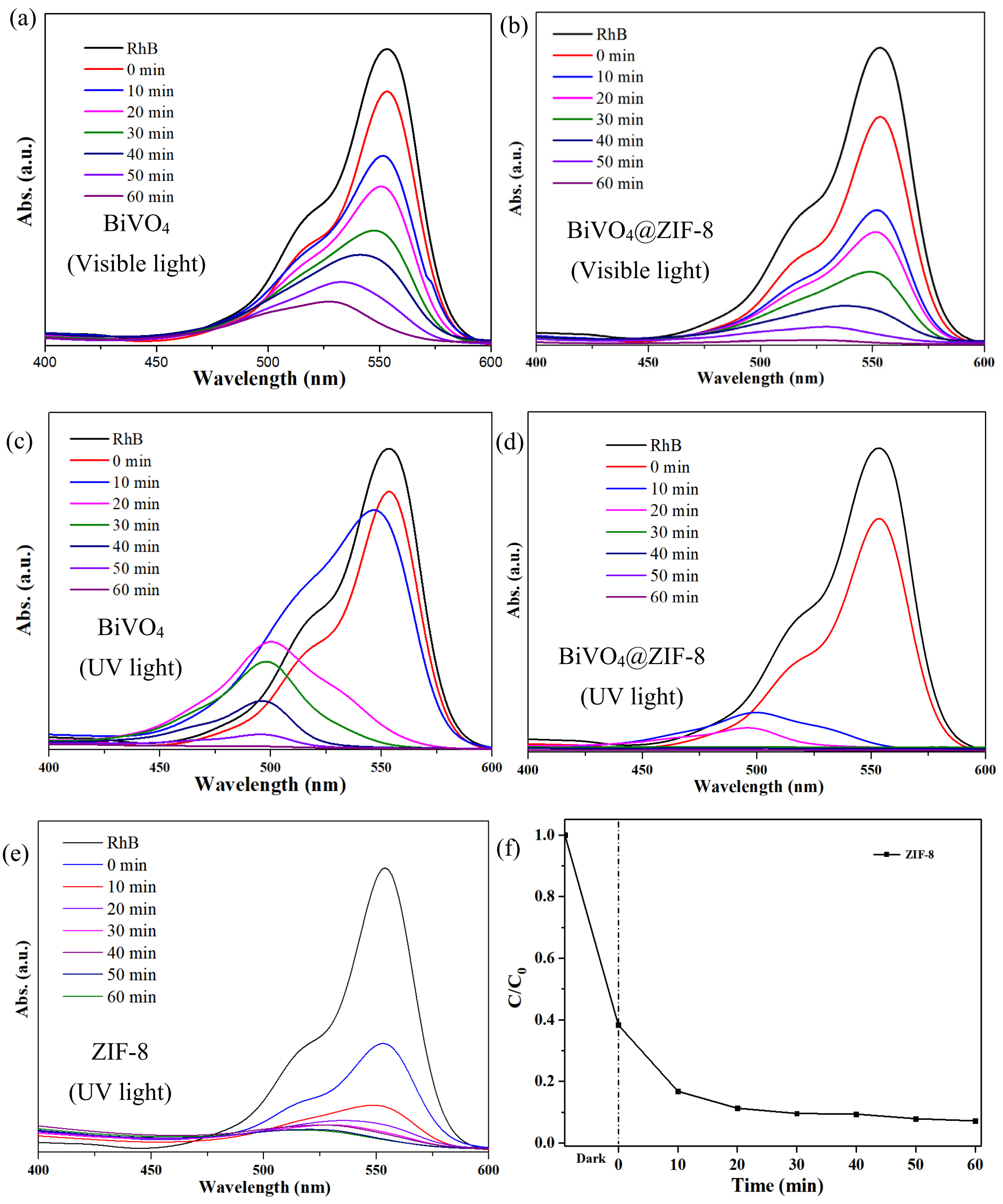

Figure S2. The light absorption spectra of RhB solution after photo-degraded by $BiVO_4$ (**a**) and $BiVO_4$@ZIF-8 (**b**) in visible light; $BiVO_4$ (**c**), $BiVO_4$@ZIF-8 (**d**) and pure ZIF-8 (**e**) in UV light; (**f**) The photo degradation efficiency of pure ZIF-8 under UV light.

Table S1. The performances of photocatalysts based on different $BiVO_4$ composite materials.

Photocatalyst	Morphology	Efficiency *	Ref.
Monoclinic-$BiVO_4$	Nanorods	60	[1]
Ag_2O @$BiVO_4$	Nanofiber	60	[2]
$BiVO_6$@TiO_2	Heterojunction structures	180	[3]
$AgNO_3$@$BiVO_4$	Dendritic-like	20	[4]
$BiVO_4$	Mesoporous	180	[5]
CdS@$BiVO_4$	Hollow cubes	50	[6]
ZnO@MoS_2@$BiVO_4$	Heterojunction structures	>180	[7]
ZIF-8@$BiVO_4$	Hierarchical structures	20	This work

* Time needed (minutes) for 90% degradation.

Table S2. The performances of photocatalysts based on different MOF composite materials.

Photocatalyst	Morphology	Efficiency *	Ref.
ZnO/CdS@ZIF-8	Nanorods	>120	[8]
ZIF-8@$Zn_{1-x}Ni_xO$	Nanoboxes	15	[9]
Mn_3O_4@ZIF-8	Nanoparticles	30	[10]
Bi_2S_3@ZIF-8	Nanorods	50 (Visible light)	[11]
Bi_2MoO_6@ZIF-8	Mesoporous ball	>100	[12]
ZIF-8@g-C3N4	Nanoparticles	30	[13]
ZIF-8@$BiVO_4$	Hierarchical structures	20	This work

* Time needed (minutes) for 90% degradation.

References

1. Y. Lin, C. Lu, C. Wei, Microstructure and photocatalytic performance of BiVO4 prepared by hydrothermal method, Journal of Alloys and Compounds, 781 (2019) 56-63.
2. J. Ren, Y. Zhu, Ag2O-decorated electrospun BiVO4 nanofibers with enhanced photocatalytic performance, RSC Advances, 10 (2020) 6114-6120.
3. C. Ma, M. Wei, BiVO4-nanorod-decorated rutile/anatase TiO2 nanofibers with enhanced photoelectrochemical performance, Materials Letters, 259 (2020) 126849.
4. M. Wang, W. Lu, D. Chen, J. Liu, B. Hu, L. Jin, Y. Lin, D. Yue, J. Huang, Z. Wang, Synthesis of dendritic-like BiVO4:Ag heterostructure for enhanced and fast photocatalytic degradation of RhB solution, Materials Research Bulletin, 84 (2016) 414-421.
5. W. He, X. Zhang, X. Dong, X. Zhang, C. Ma, H. Ma, Preparation of Mesoporous BiVO4 for Efficient Photocatalytic Degradation of RhB Under Illuminated Visible Light, Journal of Advanced Oxidation Technologies, 17 (2014) 33-38.
6. Y. Lin, D. Pan, H. Luo, Hollow direct Z–Scheme CdS/BiVO4 composite with boosted photocatalytic performance for RhB degradation and hydrogen production, Materials Science in Semiconductor Processing, 121 (2021) 105453.
7. M.B. Tahir, F. Shafiq, M. Sagir, M.S. Tahir, Construction of visible-light-driven ternary ZnO-MoS2-BiVO4 composites for enhanced photocatalytic activity, Applied Nanoscience, 11 (2021) 241-247.
8. R.-M. Kong, Y. Zhao, Y. Zheng, F. Qu, Facile synthesis of ZnO/CdS@ZIF-8 core-shell nanocomposites and their applications in photocatalytic degradation of organic dyes, RSC Advances, 7 (2017) 31365-31371.
9. Y. Jing, J. Wang, B. Yu, J. Lun, Y. Cheng, B. Xiong, Q. Lei, Y. Yang, L. Chen, M. Zhao, A MOF-derived ZIF-8@Zn1-xNixO photocatalyst with enhanced photocatalytic activity, RSC Advances, 7 (2017) 42030-42035.
10. L. Hu, G. Deng, W. Lu, Y. Lu, Y. Zhang, Peroxymonosulfate activation by Mn3O4/metal-organic framework for degradation of refractory aqueous organic pollutant rhodamine B, Chinese Journal of Catalysis, 38 (2017) 1360-1372.
11. Y.-H. Ding, X.-L. Zhang, N. Zhang, J.-Y. Zhang, R. Zhang, Y.-F. Liu, Y.-Z. Fang, A visible-light driven Bi2S3@ZIF-8 core-shell heterostructure and synergistic photocatalysis mechanism, Dalton Transactions, 47 (2018) 684-692.
12. Y. Xia, S.-k. Shang, X.-r. Zeng, J. Zhou, Y.-y. Li, A Novel Bi2MoO6/ZIF-8 Composite for Enhanced Visible Light Photocatalytic Activity, Nanomaterials, 9 (2019).
13. X. Liu, J. Zhang, Y. Dong, H. Li, Y. Xia, H. Wang, A facile approach for the synthesis of Z-scheme photocatalyst ZIF-8/g-C3N4 with highly enhanced photocatalytic activity under simulated sunlight, New Journal of Chemistry, 42 (2018) 12180-12187.
